# Trunk Lateral Flexor Endurance and Body Fat: Predictive Risk Factors for Low Back Pain in Child Equestrian Athletes

**DOI:** 10.3390/children7100172

**Published:** 2020-10-09

**Authors:** Antonio Cejudo, Angélica Ginés-Díaz, Olga Rodríguez-Ferrán, Fernando Santonja-Medina, Pilar Sainz de Baranda

**Affiliations:** 1 Department of Physical Activity and Sport, Faculty of Sport Sciences, Regional Campus of International Excellence “Campus Mare Nostrum”, University of Murcia, 30720 Murcia, Spain; antonio.cejudo@um.es (A.C.); psainzdebaranda@um.es (P.S.d.B.); 2 Sports and Musculoskeletal System Research Group (RAQUIS), Campus de San Javier, University of Murcia, 30720 Murcia, Spain; santonja@um.es; 3 Department of Surgery, Pediatrics, Obstetrics and Gynecology, Faculty of Medicine, Regional Campus of International Excellence “Campus Mare Nostrum”, University of Murcia, 30100 Murcia, Spain

**Keywords:** horse riding, asymmetry, equestrian injury, back pain, young equestrian athletes

## Abstract

Low back pain (LBP) is the most common overuse musculoskeletal injury suffered by child equestrian athletes (CEA). Despite this, little is known about the risk factors related to LBP in these athletes, and very limited research has been conducted on this topic. This study was designed to investigate predictive risk factors for LBP in CEA. The purposes of this research were to determine whether anthropometric, range of motion (ROM), core endurance and sagittal spinal morphotype measures are risk factors for LBP and to establish a diagnostic cutoff value for those factors associated with LBP. Nineteen CEA between the ages of 12 and 17 years were voluntarily recruited. Potential risk factors evaluated included corporal composition, lower limb ROM, core endurance and sagittal spinal measures. Associations and predictions were calculated between these risk factors and the LBP during the last 12 months. Almost half of the CEA have suffered at least one episode of LBP. Two risk factors and cutoff values were identified as predictors of LBP in CEA: having a high body fat higher than 23% (*p* = 0.01) and trunk lateral flexor endurance lower to 65 s (*p* = 0.021), body fat being the strongest predictor.

## 1. Introduction

Low back pain (LBP) is the most common overuse musculoskeletal injury experienced by equestrian athletes (EA) [1,2]. Compared to the general population and other sports, equestrian sports show some of the highest LBP prevalence, ranging from 20% to 88% [3,4,5,6,7,8,9]. Gandy, Bondi, Pigott, Smith and McDonald [10] described an LBP incidence in EA three to five times greater than in the general population. Recreational EA show higher incidence of LBP than professional EA [8]. However, no differences were observed in LBP prevalence values between classical dressage and horse jumping equestrian modalities [10,11].

The presence of LBP negatively affects the rider’s performance because it causes distraction in 50% of EA [3]. In addition, LBP contributes to earlier onset of fatigue [3,5], which may potentially increase the risk of a grave or lethal fall [5]. 

The high prevalence of elite EA experiencing LBP is a consequence of the biomechanical demands for equestrian sports [2,4,9]. Equestrian riding involves large and repetitive compressive mechanical forces, which are mitigated and absorbed by the vertical axis of rider’s body, especially the lumbo–pelvic–hip complex [2,8,12]; moreover, “sitting trot” produces both trunk hyperflexion and hyperextension movements [13]. EA presented higher values for lumbar curvatures in the analyzed standing, slump sitting and trunk bending positions [11]. In addition, horse jumping modality showed increased risk of injury to the lumbar intervertebral disk due to increased compressive forces caused by the horse landing and trunk forward bending position [9]. These mechanical forces and sagittal spinal morphotype are therefore considered to be the major contributors to back pain. The LBP in EA is related to damage affecting the musculature, ligaments, disks, and vertebrae (spondylolysis and spondylolisthesis) in the lumbar spine [13].

Different causes have been suggested for LBP, including a decreased trunk range of motion (ROM) [4], reduced rider stability/balance [12] and an asymmetric posture [3,4]. Determining the modifiable risk factors for LBP is a highly valuable tool to establish an efficient prevention program; for example, hippotherapy, which is a program to improve the postural alignment and the balance of head and trunk in children [14], should take into account these risk factors to increase the efficiency of the intervention. Some studies have suggested the modifiable factors such as the body composition [9], lack of muscle flexibility [4], core endurance deficit [6,8], asymmetric hip/pelvic disposition [4,5,13] and sagittal spinal misalignment [8,11] may be related to LBP in EA. However, the risk factors for LBP among EA have not been extensively investigated [10]. The aims of this research regarding child EA were to determine whether corporal composition, flexibility, core endurance and sagittal spinal curvatures measures are predisposing factors for LBP (I) and to perform a predictive injury study to establish diagnostic cutoff values for those factors associated with LBP (II). We hypothesize that the excess corporal composition, limited muscle flexibility, muscle weakness, asymmetry and sagittal spinal curvatures misalignment are correlated to LBP and may be predictors of LBP in child EA. 

## 2. Materials and Methods

### 2.1. Experimental Design

This investigation was performed on child equestrian athletes (CEA) that displayed (n = 8) or not (n = 11) LBP during the last 12 months. The current retrospective cohort considered demographic data, training regime, anthropometric traits, sagittal spinal curvatures, hip ROM and trunk muscle endurance as potential risk factors and predictive parameters for LBP in young EA. The outcome measures were conducted at the end of the competition phase of the year 2017. All testing was performed in the Equestrian Technical Centre of the Region of Murcia (Murcia, Spain). 

The recruitment of riders took place on the first day of the technical training camp. Both the questionnaire and the assessment of the potential predictive risk factors for LBP were conducted on the same day (the second day of the technical training camp). Subsequently, data were analyzed, and cutoff values were established for those variables correlated to LBP in order to identify EA with high likelihood of LBP.

### 2.2. Participants (Sample of Equestrian Athletes)

Nineteen CEA (8 males and 11 females) between the ages of 12 and 17 years (14.7 ± 1.9 y) were voluntarily recruited in a technical training camp (Table 1). The participants were the CEA of the Murcia Regional Team, who competed in the modalities of classical dressage and show jumping. The training volume defined as “training hours during the last 12 months” was calculated using the following formula: training hours × day × weekly training days × 4 weeks per month × months per year [15,16,17]. None of the participants were involved in a systematic and specific physical training program in the last 12 months. The athletes only participated in practical physical education sessions (two 60 min sessions per week).

Orthopedics problems affecting the lower limb or lower back in the last two weeks that could affect the CEA’s movement competency, anthropometric traits (weight, height, body mass index, and body fat), sagittal spinal curvatures, hip ROMs and trunk muscle endurance were considered as exclusion criteria.

Before participation, written informed consent was obtained from the parents/legal tutors and CEA following the set procedures, and they were approved by the Ethics and Research Committee of the University of Murcia (Spain) (ID: 1920/2018). The research was conducted according to the Declaration of Helsinki (1975) for studies involving human subjects. Participants were also informed that they were free to withdraw from the study at any time. The power of the sample size for this study was analyzed as described in the Statistical Analysis section.

### 2.3. Examiners

Five examiners performed data collection for this investigation. Examiners were certified as Physical Activity and Sports Sciences examiners with five or more years of experience in evaluating musculoskeletal conditions. All measurements of a specific parameter were always made by the same examiners. A double-blind study was conducted (2 testing sessions, 24 h apart) before the measurements to establish the intraexaminer reliability with 12 participants, and intraclass correlation coefficients (ICC) greater than 0.88 (anthropometric traits, 0.98 to 0.99; sagittal spinal curvatures, 0.92 to 0.94; hip ROM, 0.90 to 0.96; trunk muscle endurance 0.88 to 0.92) were obtained for all variables.

### 2.4. Interview Questionnaire

Before the assessment session, CEA completed a questionnaire about their demographic data (age, height, body mass, body mass index and body fat percentage), sport-related background (equestrian sport modality, federative category, current competitive level, dominant limb (defined as the predominant foot used for kicking a ball three times)) and systematic training workload (equestrian sport experience, days of training per week, and training hours per week). The information in the questionnaires was cross-referred with the trainer and parents in order to increase the objectivity. The anthropometric measurements included in the questionnaire (body mass, body height, body mass index and body fat percentage) were assessed by the examiners. Subsequently, the CEA were asked if they had experienced LBP for longer than 1 week or whether they did not attend at least three days of training due to LBP [18] within the last 12 months. This information was used to determine the CEA with previous history of LBP. Those CEA that experienced LBP during the sport practice or just after riding were also considered as CEA with history of LBP. The period of 1 week for LBP duration [19] was chosen to exclude simple “delayed onset muscle soreness” that may last for a few days. Similarly, the CEA with a history of previous spine surgery were excluded of this study.

### 2.5. Assessment of Predictive Risk Factors for Lower Back Pain

One familiarization pre-evaluation session was performed one week before the evaluation. The purpose of this familiarization session for the CEA was to show the correct technical execution of each test.

**Figure 1:**
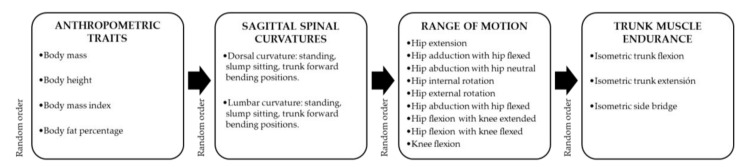
Assessment schedule of potential risk factors for lower back pain in the evaluation session.

All the CEA were asymptomatic at the time of the evaluation session. The CEA were examined wearing sports clothes and without shoes. The risk factors under study were classified in four categories (Figure 1) that were evaluated always according to the following temporal order: anthropometry traits, sagittal spinal curvatures, hip and knee ROMs and muscle endurance. However, measurements performed within each category were performed in a random order (Figure 1). All these parameters were assessed by experienced examiners. Two measurements were taken of each parameter, except for the trunk muscle endurance, which was measured only once. The mean score for each test was used in the statistical analysis. The CEA were allowed to rest for 60 s between repetitions, between evaluation of both corporal sides and for 3 min between tests.

Anthropometric traits (body mass, body height, body mass index and body fat percentage) were measured using a mobile stadiometer (Seca 213; Seca Ltd., Hamburg, Germany) and Tanita-305 body fat analyzer (Tanita Corp., Tokyo, Japan). A correction of 0.5 kg was made for the weight of the clothes. From weight and height, the body mass index (BMI) was calculated as weight/height squared (Kg/m^2^). 

Sagittal spinal curvatures (dorsal and lumbar) were examined in a relaxed standing position (SP), in a slump sitting position (SSP), as well as in trunk forward bending position (TFB) following the methodology of Santonja-Medina et al. [20], which has been previously used in other studies [20,21,22].

The maximum passive 9 hip and knee ROMs of the dominant and non-dominant limb were assessed by using the ROM-SPORT battery [15,23,24,25]. Both sagittal spinal curvatures and ROM were measured based on inclinometer techniques (ISOMED Unilevel inclinometer, Portland, OR, United States).

Trunk muscle (flexors, extensors, and lateral flexor) endurance was tested with the field tests of isometric trunk flexion (ITF) [26], isometric trunk extension (ITE) [27,28], dynamic trunk flexion-rotation (DTFR) [29] and isometric side bridge endurance for dominant and non-dominant sides [27]. A chronometer and metronome were used to control the time and execution speed of each test repetition, respectively.

### 2.6. Statistical Analysis

The normality of the distribution for each variable was examined using the Shapiro–Wilk normality tests, and homogeneity of variance was verified with a Levene test between the male group and the female group. 

Descriptive statistics including means and standard deviations were calculated for hip and knee ROM and trunk lateral flexor endurance measures separately by lower limb dominance (dominant and non-dominant). 

Data were analyzed using independent sample the Mann–Whitney U test to examine possible differences in demographic and sport variables according to sex. The Wilcoxon signed rank test was used to evaluate the asymmetry between body sides. In addition, Cohen’s effect size was calculated for all trunk lateral flexor and ROMs results. The magnitude of the effect size was classified as previously described by Hopkins et al. [30] as trivial (<0.2), small (0.2 to 0.59), moderate (0.6 to 1.19), large (1.20 to 2.00), very large (2.00 to 3.99) or extremely large (>4.0). Asymmetry was considered when the magnitude of the effect size was moderate, which is stablished as the minimum level of a relevant effect with practical application [30], or higher than moderate. 

The Mann–Whitney U test was used to compare the continuous variables (anthropometric characteristics, sport-related background and training regimen variables, ROMs and asymmetry) between the CEA with a previous history of LBP (CEA-LBP) and the asymptomatic equestrian athletes (CEA-A); Additionally, Cohen’s effect size was calculated for all ROM results, and the magnitudes of the effect were classified as described above. 

The relationship between the independent variables and the dependent variable was calculated by backward stepwise binary logistic regression (forward selection (conditional), inclusion probability: *p* ≤ 0.05, elimination probability: *p* ≤ 0.10) with odds ratio (OR) analysis being applied as in previous studies [15,31,32] for estimating the simultaneous effects of several predictors instead of relative risk estimates [33]. Effect sizes for the OR were defined as follows: small effect OR = 1 to 1.25, medium effect OR = 1.25 to 2 and large effect OR ≥ 2 [34].

To determine whether it was possible to find predictive cutoff values for those variables (risk factors) associated with LBP that could be used for pointing out individuals at high risk for LBP, receiver operating characteristic (ROC) curves were calculated. The area under the ROC curve represents the probability that a selection based on the risk factor for a randomly chosen positive case will exceed the result for a randomly chosen negative case. The area under the curve can range from 0.5 (no accuracy) to 1.0 (perfect accuracy). If it is found to be statistically significant (*p* < 0.05), it means that using the risk factor as a determinant is better than guessing. Since the ROC curve plots sensitivity against 1 minus specificity, the coordinates of the curve can be considered possible cutoff points. The best cutoff values were chosen using the Youden’s index.

Among the CEA-LBP, Pearson’s chi-squared test was used to examine the existence of a relationship between the variables associated with LBP classification (normal and high risk) and LBP. 

In order to calculate the power of the sample size, a post hoc power analysis was conducted using the software package, G*Power 3.1.9.4 [35]. 

Analysis was performed using the Statistical Package for the Social Sciences (SPSS) version 24 software (SPSS Inc, Chicago, IL, USA). Statistical significance was set at *p* < 0.05. Results were reported as mean ± SD and 95% confidence interval (CI).

## 3. Results

Statistical analysis revealed that data did not have a normal distribution. The sample was homogeneous in potential confounding variables, except in trunk lateral flexor endurance (*p* = 0.006) and stature (*p* = 0.042) according to sex. 

Descriptive values of sagittal spine curvature, hip and knee ROM and trunk muscle endurance parameters by limb dominance are shown in Table 2. In the 19 CEA, the Mann–Whitney U test showed higher (*p* ≤ 0.040; Cohen’s d ≤ 0.442 (small)) values of hip extension test (HE), hip abduction with hip neutral test (HAB), hip external rotation test (HIR) and side bridge tests for the dominant limb than those recorded for the non-dominant limb. On the contrary, hip adduction with hip flexed test (HAD-HF) and hip flexion with knee extended test (HF-KF) values were higher (*p* ≤ 0.039; Cohen’s d ≤ 0.527 (small)) for the non-dominant limb.

Sample sizes of 8 (athletes with a previous history of LBP) and 11 (asymptomatic equestrian athletes) participants, an alpha level of *p* < 0.05, effect size (Cohen’s d) and the Mann–Whitney U test were used for the power analyses. The predictive variables (body fat and isometric side bridge endurance (ISBE)) obtained in this this study (Table 3), obtained a statistical power of 0.83 for body fat and 0.72 for trunk lateral flexor endurance.

Of the 19 CEA involved in this study, 8 CEA experienced LBP at least once during the last 12 months and were considered as EA with a previous history of LBP (CEA-LBP). Asymptomatic equestrian athletes (CEA-A) and CEA-LBP had similar training volume during the period of study. 

There were significant differences effect size between the CEA-LBP group and the CEA-A group for body fat (CEA-A: 19.3% vs. CEA-LBP: 26.1%, *p* = 0.010, Cohen’s d = −1.2668 (large effect sizes)), hip total rotation (HTR) (CEA-A: 115.8° vs. CEA-LBP: 125.0°, *p* = 0.043, Cohen’s d = −0.8971 (moderate effect sizes)), isometric side bridge endurance for non-dominant (ISBE_ND) (CEA-A: 79.3 s vs. CEA-LBP: 52.7 s, *p* = 0.039, Cohen’s d = 1.0203 (large effect sizes)) and ISBE (CEA-A: 85.9 s vs. CEA-LBP: 58.2 s, *p* = 0.021, Cohen’s d = 1.076 (large effect sizes)). The group of EA-LBP had an increased range of 6.8% and 9.2° in body fat and HTR and a decreased range of 26.6 s and 27.7 s in ISBE_ND and ISBE, respectively compared to CEA-A (Table 3).

A first stepwise logistic regression analysis (enter method) with the possible risk factors for LBP of Table 4 (body fat, HTR and ISBE) achieved high classification accuracy (84.2%) for CEA with or without a previous history of LBP (sensibility = 87.5%; specificity = 81.8%). However, this model did not find causal relationships between predictive variables (body fat (*p* = 0.203), HTR (*p* = 0.267) and ISBE (*p* = 0.313)) and previous history of LBP. Stepwise logistic regression analysis (forward selection (conditional)) showed that of the potential risk factors for LBP of Table 4 (body fat, HTR and ISBE) entered into the model, body-fat and ISBE showed medium (OR = 1.297 (medium), 95% CI = 1.005 to 1.673, *p* = 0.045) and small (OR = 1.048 (small), 95% CI = 0.910 to 1.001, p = 0.055) predictors of previous history of LBP in the 19 CEA. In addition, the analysis of the frequencies showed 70% of successful cases in CEA-LBP who were categorized with high body fat (cutoff ≥ 23%) and 66.7% of successful cases in CEA-LBP who were categorized with low ISBE (cutoff ≤ 65 s), according to the present study. None of the other intrinsic factors imposed a significant relative risk for LBP (*p* > 0.05).

**Figure 2:**
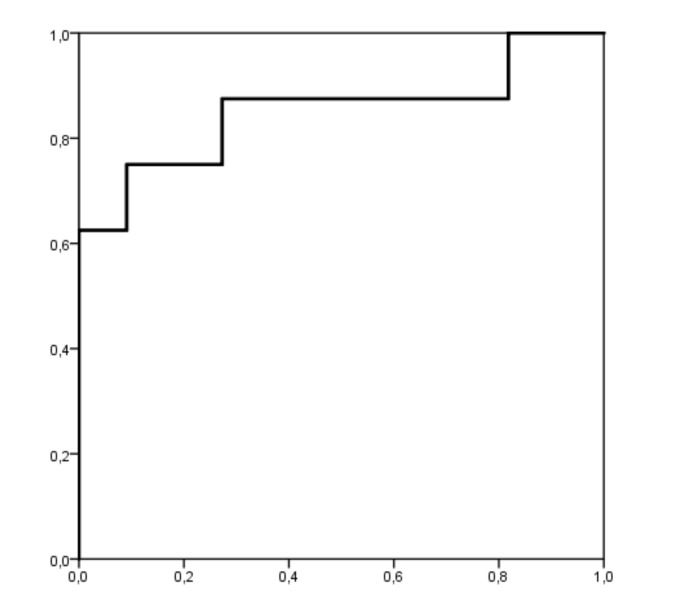
Receiver operating characteristic (ROC) curve analysis for body fat as a risk factor for lower back pain. The area under the curve is 0.852 (*p* = 0.01); the coordinates represent a possible cutoff point in body fat (the optimal cutoff point was 23%).

**Figure 3:**
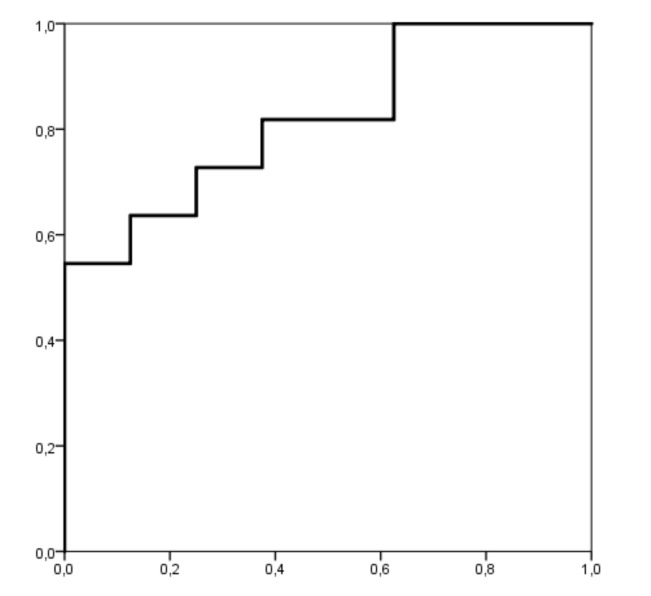
Receiver operating characteristic curve (ROC) analysis for ISBE as a risk factor for lower back pain. The area under the curve is 0.818 (p = 0.021); the coordinates represent a possible cutoff point in ISBE (the optimal cutoff point was 65 s).

Body fat and ISBE showed a good accuracy of the predictive model for CEA-LBP [36]. The area under the ROC curves for body fat and ISBE was 0.852 and 0.818, respectively (Figure 2 and Figure 3), being statistically significant (body fat (*p* = 0.01, standard error: 0.102, 95% confidence interval: 0.651 to 1.000) and ISBE (*p* = 0.021, standard error: 0.097, 95% confidence interval: 0.629 to 1.000)). Using the coordinates of the curves, the angles of body fat and ISBE that most accurately identified individuals at risk for LBP were 23% (sensibility of 0.875 and 0.273 specificity) and 65 s (sensibility of 0.818 and 0.375 specificity), respectively.

Finally, Pearson’s chi-square test showed that high body fat (≥23%) tended to be associated with having CEA-LBP (X^2^_(19)_ = 6.739; *p* = 0.009; η2 = 0.596). When the relative risk was estimated, CEA with greater body fat (≥23%) had 18.7 times higher risk of LBP (95% CI = 1.563 to 222.926) than CEA with a normal body fat (<23%). Pearson’s chi-square test showed that lower ISBE (≤65 s) tended to be associated with LBP (X^2^_(19)_ = 2.170; p = 0.141; η2 = 0.338). When the relative risk was estimated, EA with lower ISBE (≤65 s) had 4.5 times higher risk of CEA-LBP (95% CI = 0.570 to 35.519) than CEA with a normal ISBE (>65 s).

## 4. Discussion

### 4.1. Risk Factors for the Development of Low Back Pain in CEA

To the best of our knowledge, this is the first report that investigates and identifies risk factors for LBP in CEA. The current study reports significant differences between CEA-LBP and CEA-A with respect to the percentage body fat, HTR, ISBE_ND and ISBE in the descriptive analysis. Interestingly, CEA-LBP had lower values in ISBE and ISBE_ND and higher percentage body fat and HTR than CEA-A. Some of these results were confirmed by the regression model, which demonstrated that the main predisposing factors for history of LBP in CEA were a body fat higher than 23% and ISBE lower than 65 s (trend towards statistical significance, *p* = 0.055). This expected result is the main finding of our study and will be highly useful in the prevention of LBP in these athletes.

In present study, CEA-LBP reported a body fat of 6.8% more than CEA-A. The mean values of percentage body fat for CEA-LBP reported in this study are similar to those reported previously (range from 23.4% to 28.6%) in collegiate EA [37], adolescent female EA [38] and in collegiate female EA [39,40]. The high body fat percentage reported in EA reflects a worse physical conditioning when compared to other groups of athletes [41,42]. 

It is known that athletes with higher body composition in term body mass, body fat, waist circumference and body mass index are at higher risk for LBP in some sports [18] such as adolescent rhythmic gymnasts [41], collegiate gymnasts [42], collegiate and adult judo athletes [43,44,45], young ice hockey and soccer players [46] and young golfers [47]. In this sense, the present study also demonstrates that body composition is related to the incidence of LBP in this case in EA. Our results are consistent with Kraft et al. [9], Kujala et al. [46], Kujala et al. [48], and Burdorf, Van Der Steenhoven and Tromp-Klaren [49], who did not find a significant association between other anthropometric traits such as stature and body mass index with LBP. Evans, Refshauge, Adams and Aliprandi [50] found surprisingly higher body mass index values in golfers without LBP that golfers with LBP.

Several studies reported poor endurance test scores (trunk extensor endurance, trunk flexor endurance, and trunk lateral flexor endurance) related to LBP in athletes [48,50]. In this study, ISBE was 27.7 s shorter in CEA-LBP than in CEA-A. The cutoff score with the greatest discriminatory power for prognostic screening were those obtained from ISBE (≤65 s). These results are in accordance with those reported by Evans et al. [50] in young golfers. Specifically, Evans, Refshauge, Adam and Aliprandi [50] observed that golfers with a right-side deficit of >12.5 s on ISBE reported more frequent episodes of LBP. In our study, CEA-LBP showed a non-dominant side ISBE deficit of 10.9 s. (the non-dominant side showing a deficit of 13.1 s). It appears that EA may strengthen the trunk lateral flexors to support the repetitive biomechanical demands of the modalities of classical dressage and show jumping. In the sense, Lewis and Kennerley [3] recommend that EA need to have strong abdominal and back musculature to maintain the lumbar pelvic hip complex around the central longitudinal axis in order to maintain the correct riding position. Core endurance deficiency may have a negative impact on the rider maintaining an effective dressage position [3]. Terada et al. [51] also reported the need for core endurance due to the long periods in which riders maintain muscles in tonic contraction to control posture during trot. In addition, EA-LBP and core fatigue can reduce the rider’s ability to synchronize with the horse’s movement [52] and therefore increases lumbar compressive and mechanical loads [9]. On the contrary, no significant relation was found between LBP and trunk flexor and extensor endurance in this study; this finding is in accordance with those previously reported for other sports [26,48,50]. The CEA in this study show high values for trunk flexor and extensor endurance as adaptations of the training in both groups; it seems that this do not determine the cause of LBP. However, these trunk muscles present a greater demand in the anterior and posterior pelvic tilt to accompany the different movements of the horse (walk, trot, canter, and gallop). In addition, it is possible suffering from LBP in the previous year has a stronger impact on trunk proprioception and stability, which are the main functions of trunk lateral flexors, rather than on current trunk muscle endurance. Another point to consider is that even CEA-LBP could have recovered completely from their pain and improved their fitness status until the day of the assessments.

In the present study, significant asymmetry was observed in HE, HAD-HF, HAB, HIR, HF-KF and ISBE, but these results are not considered relevant in sports practice. More specifically, higher values are observed on the dominant side than on the non-dominant side in both groups (CEA-A and CEA-LBP). For ISBE, among the risk factors of LBP, side-to-side differences (the dominant side hold-time should be divided by the non-dominant side hold-time) are superior in CEA-LBP (ratio = 1.21) than in EA-A (ratio 1.17). A value greater than 1.05 or less than 0.95 indicates a significant degree of asymmetry [53]. However, this type of asymmetry has not been a predictor of LBP in CEA in this study. However, significant asymmetries of ROM and ISBE may contribute to the frequent asymmetric posture of EA during horseback riding. 

In this sense, the EA’s asymmetric posture during horseback riding has been considered as an important contributing factor to back pain [54]. Unilateral lumbopelvic hip drop or collapse of EA increased pressure under the saddle on the same side or on the opposite side [4]. Frontal plane asymmetric hip/pelvic disposition-modified tension patterns within the musculoskeletal system—which stabilizes the pelvis due to side-to-side differences in the mechanical loads transmitting through it—increase the potential of developing asymmetry postural [55]. Long-term repeated application of asymmetrical forces over a series of training events decreases the efficiency of horse learning and welfare, reduces rider stability/balance, and subsequently contributes to injury and pain to the back and limbs of both the horse [12,56] and rider [5,12,13,57,58]. Several authors have shown that frontal plane hip/pelvic postural asymmetry has been associated with the repetitive demands of equestrian sports [4], high competitive levels [4,56], years of riding experience [4,58], pain avoidance [4], leg length discrepancies [57,58], and lateral bending and hip extension ROM [4,59]. 

Finally, it has to be noted that the results of the present investigation should not be generalized, as only 19 young athletes from a specialized sample took part in the study. For this reason, future research should considerer a larger sample size where CEA from different technical training camps participate.

### 4.2. Practical Considerations

The first step to reduce LBP in CEA is the evaluation of the modifiable risk factors for LBP (for body composition and trunk muscle endurance) to identify athletes with higher risk of injury. According to the results achieved in this study, the CEA need to supplement their sport practice with aerobic and strength regimens, specifically sport-specific core endurance, stability and strength (lumbopelvic hip region) and postural training in order to reduce LBP incidence. 

It has been proposed that at least 180 min per week should be dedicated to aerobic resistance exercises in the form of three 60 min sessions of moderate intensity for a child or adolescent [60]. With regard to strength, an 8-week training program (3 days/week, 8 exercises, 3 sets, 3–15 reps and 90–120 s rest) significantly improves body composition (% fat body and mass index) and increases strength and power in children who are overweight or obese [59]. In addition, it has been demonstrated [61] that training the trunk musculature twice per week during a 10-week period with a relatively simple floor exercise protocol is an effective stimulus to improve trunk endurance. Therefore, to reduce LBP, all these recommendations should be considered when designing training programs for CEA.

## 5. Conclusions

In this study, almost half of the child equestrian athletes suffered at least one episode of LBP within the last 12 months. Two risk factors and cutoff values were identified as predictors of LBP in this specific sample of child equestrian athletes and having a high body fat greater than 23% and ISBE lower or equal to 65 s, body fat being the strongest predictor.

## Figures and Tables

**Table 1:** Demographic and sport data for equestrian athletes of the Murcia Regional Team.

Variable	Male (n = 8)	Female (n = 11)	*p*-Value	Total (n = 19)
Age (years)	13.9 ± 1.8	15.3 ± 1.9	0.81	14.7 ± 1.9
Stature (cm)	159.3 ± 14.3	160.8 ± 5.5	0.93	160.2 ± 9.9
Body mass (kg)	52.9 ± 13.8	53.2 ± 9.5	0.87	53.1 ± 11.1
Body mass index (kg/m2)	20.6 ± 2.9	20.5 ± 3.2	0.62	20.5 ± 3.0
Body fat (%)	19.4 ± 4.2	24.2 ± 6.9	0.90	22.2 ± 6.2
Riding experience (years)	5.8 ± 1.7	7.1 ± 2.5	0.16	6.5 ± 2.2
Hours of training per week (h)	7.5 ± 4.0	6.5 ± 4.7	0.37	6.9 ± 4.3
Training hours last 12 month (h)	324.5 ± 162.8	280.0 ± 182.8	0.37	298.7 ± 171.4

**Table 2:** Passive maximum lower limb range of motion and trunk lateral flexor endurance values for the 19 child equestrian athletes.

Variable	Dominant Limb	Non-Dominant Limb	*p*-Value	Cohen’s d
HE (iliopsoas)	12.3 ± 5.5°	11.3 ± 4.7°	0.040	0.195 Trivial
HAD-HF (piriformis)	25.5 ± 3.7°	27.3 ± 3.1°	0.006	−0.527 Small
HAB (adductors)	38.0 ± 4.4°	36.2 ± 3.7°	0.026	0.442 Small
HIR (external rotators)	56.1 ± 9.5°	54.2 ± 8.1°	0.028	0.215 Small
HER (internal rotators)	64.1 ± 8.5°	64.9 ± 7.2°	0.462	−0.101 Trivial
HAB-HF (M. adductors)	59.8 ± 7.3°	58.6 ± 6.0°	0.198	0.179 Trivial
HF-KE (hamstrings)	70.3 ± 7.4°	72.0 ± 6.9°	0.072	−0.237 Small
HF-KF (gluteus maximus)	135.8 ± 6.1°	137.7 ± 5.7°	0.039	−0.321 Small
KF (quadriceps)	129.7 ± 8.9°	128.6 ± 8.7°	0.206	0.125 Trivial
ISBE (trunk lateral flexors)	80.3 ± 32.7 s	68.2 ± 28.8 s	0.024	0.392 Small

M: monoarticular; HE: hip extension test; HAD-HF: hip adduction with hip flexed test; HAB: hip abduction with hip neutral test; HIR: hip internal rotation test; HER: hip external rotation test; HAB-HF: hip abduction with hip flexed test; HF-KE: hip flexion with knee extended test; KF: knee flexion test; HF-KF: hip flexion with knee flexed test; ISBE: isometric side bridge endurance. The magnitude of the effect size of the pooled standardized mean differences (SMD) was interpreted as trivial or no effect if SMD < 0.2; small if SMD = 0.2 to 0.59; moderate if SMD = 0.6 to 1.19; large if SMD = 1.20 to 2.00; very large if SMD = 2.00 to 3.99; and extremely large if SMD = greater than 4.00 [30].

**Table 3:** Comparative analysis between equestrian athletes with a previous history of LBP and asymptomatic ones. Data are expressed as mean ± standard deviation.

Variables	CEA-A (n = 11)	CEA-LBP (n = 8)	*p*-Value	Effect Sizes Cohen’s d (Qualitative Inference)
Age (years)	14.9 ± 2.0	14.0 ± 2.7	0.702	0.3889 (Small)
Body mass (kg)	53.0 ± 10.8	53.2 ± 12.3	0.934	−0.0175 (Trivial)
Height (cm)	162.7 ± 7.4	156.6 ± 12.2	0.282	0.6309 (Moderate)
BMI (kg/m2)	19.9 ± 2.7	21.4 ± 3.4	0.457	−0.4987 (Small)
Body fat (%)	19.3 ± 4.3	26.1 ± 6.6	0.010	−1.2668 (Large)
Years of experience (y)	6.9 ± 2.0	6.5 ± 2.0	0.557	0.2 (Small)
Training hours per week (h)	8.0 ± 4.4	5.1 ± 2.4	0.089	0.7818 (Moderate)
Training hours last 12 month (h)	352.0 ± 193.8	225.5 ± 106.3	0.089	0.7735 (Moderate)
Standing position (TC)	40.9 ± 8.0	39.5 ± 9.4	0.934	0.1627 (Trivial)
Standing position (LC) (°)	39.1 ± 7.8	40.8 ± 11.7	0.648	−0.1771 (Trivial)
Slump sitting (TC) (°)	42.7 ± 7.4	33.0 ± 14.5	0.171	0.89 (Moderate)
Slump sitting (LC) (°)	12.2 ± 17.3	15.3 ± 8.3	0.679	−0.2168 (Small)
Trunk forward bending (TC) (°)	52.5 ± 8.9	48.3 ± 14.7	0.868	0.3607 (Small)
Trunk forward bending (LC) (°)	28.4 ± 8.3	30.5 ± 9.9	0.587	−0.2335 (Small)
HE (iliopsoas) (°)	10.3 ± 3.1	13.9 ± 6.5	0.212	−0.7498 (Moderate)
HAD-HF (piriformis) (°)	25.9 ± 2.8	27.0 ± 3.8	0.647	−0.3385 (Small)
HAB (adductors) (°)	35.7 ± 4.1	39.0 ± 2.3	0.056	−0.95 (Moderate)
HIR (external rotators) (°)	53.1 ± 9.4	58.0 ± 7.0	0231	−0.5769 (Small)
HER (internal rotators) (°)	62.7 ± 8.9	67.0 ± 4.8	0.222	−0.5742 (Small)
HTR (hip rotators) (°)	115.8 ± 12.7	125.0 ± 5.0	0.043	−0.8971 (Moderate)
HAB-HF (monoarticular adductors) (°)	57.8 ± 7.3	61.1 ± 4.7	0.145	−0.5189 (Small)
HF-KE (hamstring) (°)	69.3 ± 5.2	73.8 ± 8.3	0.227	−0.6763 (Moderate)
HF-KF (gluteus maximus) (°)	136.6 ± 5.5	136.9 ± 6.0	0.804	−0.0525 (Trivial)
KF (quadriceps) (°)	126.8 ± 7.1	132.4 ± 9.8	0.116	−0.6732 (Moderate)
Trunk flexion (trunk flexors) (s)	230.5 ± 102.7	182.7 ± 110.9	0.372	0.4503 (Small)
Trunk extension (trunk extensors) (s)	287.2 ± 42.2	260.1 ± 81.0	0.363	0.4426 (Small)
ISBE _D (trunk lateral flexors) (s)	92.4 ± 30.7	63.6 ± 28.7	0.083	0.9634 (Moderate)
ISBE_ND (trunk lateral flexors) (s)	79.3 ± 29.2	52.7 ± 20.8	0.039	1.0203 (Moderate)
ISBE (trunk lateral flexors) (s)	85.9 ± 27.5	58.2 ± 23.0	0.021	1.076 (Moderate)
DTFR (trunk flexors/lateral flexors) (s)	63.1 ± 9.3	59.8 ± 20.6	0.563	0.2197 (Small)

LBP: lumbar back pain; CEA-LBP: child equestrian athletes with a previous history of LBP; CEA-A: asymptomatic child equestrian athletes; TC: thoracic curve; LC: lumbar curve; HE: hip extension test; HAD-HF: hip adduction with hip flexed 90° test; HAB: hip abduction with hip neutral test; HIR: hip internal rotation test; HER: hip external rotation test; HAB-HF hip abduction with hip flexed 90° test; HF-KE: hip flexion with knee extended test; HF-KF hip flexion with knee flexed test; ITF: isometric trunk flexion; ITE: isometric trunk extension; ISBE: isometric side bridge endurance; ISBE_D: isometric side bridge endurance (dominant side); ISBE_ND: isometric side bridge endurance (non-dominant side); DTFR: dynamic trunk flexion-rotation. Effect size (standardized mean difference (SMD)) were was interpreted as trivial or no effect (SMD < 0.2), small (SMD = 0.2 to 0.59), moderate (SMD = 0.6 to 1.19), large (SMD = 1.20 to 2.00), very large (SMD = 2.00 to 3.99) and extremely large (SMD > 4.00) [30].

**Table 4:** Relative frequencies and logistic regression results for lower back pain for the 19 equestrian athletes.

Risk Factors	History Last 12 Months		OR *	SE	95% CI	*p*-Value
Body fat	CEA-A	CEA-LBP	1.297 Medium	0.130	1.005 to 1.673	0.045
<23%	88.9%	11.1%				
≥23%	30.0%	70.0%				
ISBE	CEA-A	CEA-LBP	1.048 Small	0.240	0.910 to 1.001	0.055
>65 s	69.2%	30.8%				
≤65 s	33.3%	66.7%				

CEA-A: asymptomatic child equestrian athletes; CEA-LBP: child equestrian athletes with a previous history of lower back pain; OR: odds ratio (relative risk); SE: standard error; CI: confidence interval. * OR < 1: poor predictor of LBP; OR from 1 to 1.25: small predictor; OR from 1.25 to 2: medium predictor; OR ≥ 2: large predictor [34].

## References

[1] Ekberg J., Timpka T., Ramel H., Valter L. (2011). Injury rates and risk-factors associated with eventing: A total cohort study of injury events among adult Swedish eventing athletes. Int. J. Inj. Contr. Saf. Promot..

[2] Pugh T., Bolin D. (2004). Overuse injuries in equestrian athletes. Overuse Inj. Equest. Athletes.

[3] Lewis V., Kennerley R. (2017). A preliminary study to investigate the prevalence of pain in elite dressage riders during competition in the United Kingdom. Comp. Exerc. Physiol..

[4] Hobbs S.J., Baxter J., Broom L., Rossell L.A., Sinclair J., Clayton H.M. (2014). Posture, flexibility and grip strength in horse riders. J. Hum. Kinet..

[5] Lewis V., Baldwin K. (2018). A preliminary study to investigate the prevalence of pain in international event riders during competition, in the United Kingdom. Comp. Exerc. Physiol..

[6] Kraft C., Scharfstädt A., Yong M., Westhoff B., Urban N., Falkenhausen M., Pennekamp P. (2007). Correlation of back pain and magnetic resonance imaging of the lumbar spine in elite horse vaulters. Sport. Sport..

[7] Pilato M., Shifrin S., Bixby-Hammett D. (2007). The equestrian as an athlete: A view into injuries and incidence rates. Equest. Med. Saf. Assoc. Newsl..

[8] Dąbek J., Koczy B., Piotrkowicz J. (2015). Horse riding as a form of recreation and professional sport taking into account the spine mobility of riders-a preliminary results. Pol. Merkur. Lek. Organ Pol. Tow. Lek..

[9] Kraft C.N., Pennekamp P.H., Becker U., Young M., Diedrich O., Lüring C., von Falkenhausen M. (2009). Magnetic resonance imaging findings of the lumbar spine in elite horseback riders: Correlations with back pain, body mass index, trunk/leg-length coefficient, and riding discipline. Am. J. Sports Med..

[10] Gandy E.A., Bondi A., Pigott T., Smith G., Mcdonald S. (2018). Measurement of hip flexion and pelvic rotation in horse riders using imus investigation of the use of inertial sensing equipment for the measurement of hip flexion and pelvic rotation in horse riders. Comp. Exerc. Physiol..

[11] Ginés-Díaz A., Martinez-Romero M., Cejudo A., Aparicio-Sarmiento A., Sainz de Baranda P. (2019). Sagittal spinal morphotype assessment in dressage and show jumping riders physical. J. Sport Rehabil..

[12] Nevison C.M., Timmis M.A. (2013). The effect of physiotherapy intervention to the pelvic region of experienced riders on seated postural stability and the symmetry of pressure distribution to the saddle: A preliminary study. J. Vet. Behav. Clin. Appl. Res..

[13] Quinn S., Bird S. (1996). Influence of saddle type upon the incidence of lower back pain in equestrian riders. Br. J. Sports Med..

[14] Martín-Valero R., Vega-Ballón J., Perez-Cabezas V. (2018). Benefits of hippotherapy in children with cerebral palsy: A narrative review. Eur. J. Paediatr. Neurol..

[15] Cejudo A., Moreno-Alcaraz V.J., Izzo R., Santonja-Medina F., Sainz de Baranda P. (2020). External and total hip rotation ranges of motion predispose to low back pain in elite Spanish inline hockey players. Int. J. Environ. Res. Public Health.

[16] Sainz de Baranda P., Santonja-Medina F., Rodríguez-Iniesta M. (2010). Tiempo de entrenamiento y plano sagital del raquis en gimnastas de trampolín. Rev. Int. Med. Cienc. Act. Física Deport..

[17] Wojtys E., Ashton-Miller J., Huston L., Moga P. (2000). The association between athletic training time and the sagittal curvature of the immature spine. Am. J. Sports Med..

[18] Trompeter K., Fett D., Platen P. (2017). Prevalence of back pain in sports: A systematic review of the literature. Sport. Med..

[19] Vad V., Bhat A., Basrai D., Gebeh A., Aspergren D., Andrews J. (2004). Low back pain in professional golfers: The role of associated hip and low back range-of-motion deficits. Am. J. Sports Med..

[20] Santonja F., Collazo-Diéguez M., Martínez-Romero M., Rodríguez-Ferrán O., Aparicio-Sarmiento A., Cejudo A., Andújar P., Sainz De Baranda P. (2020). Classification system of the sagittal integral morphotype in children from the ISQUIOS programme (Spain). Int. J. Environ. Res. Publ. Health.

[21] Sainz-de-Baranda P., Santonja-Medina F., Rodríguez-Iniesta M. (2009). Valoración de la disposición sagital del raquis en gimnastas especialistas en trampolín. Assessment of the sagittal plane of the spine in trampoline gymnasts. RICYDE Rev. Int. Cienc. Deport..

[22] Sainz de Baranda P., Cejudo A., Moreno-Alcaraz V., Martinez-Romero M., Aparicio-Sarmiento A., Santonja F. (2020). Sagittal spinal morphotype assessment in 8 to 15 years old inline hockey players. Peer J..

[23] Sainz de Baranda P., Cejudo A., Ayala F., Santonja F. (2015). perfil óptimo de flexibilidad del miembro inferior en jugadoras de fútbol sala. Rev. Int. Med. Cienc. Act. Fis. Deport..

[24] Cejudo A., Moreno-Alcaraz V.J., Izzo R., Robles-Palazón F.J., Sainz de Baranda P., Santonja-Medina F. (2020). Flexibility in Spanish elite inline hockey players: Profile, sex, tightness and asymmetry. Int. J. Environ. Res. Publ. Health.

[25] Cejudo A. (2020). El perfil óptimo de flexibilidad en jóvenes jugadores de fútbol durante su periodo sensible del desarrollo físico. Batería ROM-SPORT. JUMP.

[26] Ito T., Shirado O., Suzuki H., Takahashi M., Kaneda K., Strax T.E. (1996). Lumbar trunk muscle endurance testing: An inexpensive alternative to a machine for evaluation. Arch. Phys. Med. Rehabil..

[27] McGill S., Childs A., Liebenson C. (1999). Endurance times for low back stabilization exercises: Clinical targets for testing and training from a normal database. Arch. Phys. Med. Rehabil..

[28] Martínez-Romero M.T., Ayala F., de Ste Croix M., Vera-Garcia F.J., Sainz de Baranda P., Santonja-Medina F., Sánchez-Meca J. (2020). A meta-analysis of the reliability of four field-based trunk extension endurance tests. Int. J. Environ. Res. Public Health.

[29] Brotons-Gil E., García-Vaquero M.P., Peco-González N., Vera-Garcia F.J. (2013). Flexion-rotation trunk test to assess abdominal muscle endurance. J. Strength Cond. Res..

[30] Hopkins W., Marshall S., Batterham A., Hanin J. (2009). progressive statistics for studies in sports medicine and exercise science. Med. Sci. Sport. Exerc..

[31] Fousekis K., Tsepis E., Poulmedis P., Athanasopoulos S., Vagenas G. (2011). Intrinsic risk factors of non-contact quadriceps and hamstring strains in soccer: A prospective study of 100 professional players. Br. J. Sports Med..

[32] Witvrouw E., Bellemans J., Lysens R., Danneels L., Cambier D. (2001). Intrinsic risk factors for the development of patellar tendinitis in an athletic population. Am. J. Sports Med..

[33] Fagerland M., Hosmer D. (2012). A generalized hosmer-lemeshow goodness-of-fit test for multinomial logistic regression models. Stata J..

[34] Coombes B., Bisset L., Vicenzino B. (2010). Efficacy and safety of corticosteroid injections and other injections for management of tendinopathy: A systematic review of randomised controlled trials. Lancet.

[35] Faul F., Erdfelder E., Lang A., Buchner A. (2007). G*Power 3: A flexible statistical power analysis program for the social, behavioral, and biomedical sciences. Behav. Res. Methods.

[36] Cortes C., Mohri M. (2004). AUC optimization vs. error rate minimization. Adv. Neural Inf. Process. Syst..

[37] Meyers M.C. (2006). Effect of equitation training on health and physical fitness of college females. Eur. J. Appl. Physiol..

[38] Alfredson H., Hedberg G., Bergström E., Nordström P., Lorentzon R. (1998). High thigh muscle strength but not bone mass in young horseback-riding females. Calcif. Tissue Int..

[39] Meyers M., Sterling J. (2000). Physical, hematological, and exercise response of collegiate female equestrian athletes. J. Sports Med. Phys. Fitness.

[40] Roberts M., Shearman J., Marlin D. (2009). A comparison of the metabolic cost of the three phases of the one-day event in female collegiate riders. Comp. Exerc. Physiol..

[41] Cupisti A., D’Alessandro C., Evangelisti I., Piazza M., Galetta F., Morelli E. (2004). Low back pain in competitive rhythmic gymnasts. J. Sports Med. Phys. Fitness.

[42] Koyama K., Nakazato K., Min S., Gushiken K., Hatakeda Y., Seo K., Hiranuma K. (2013). Radiological abnormalities and low back pain in gymnasts. Int. J. Sports Med..

[43] Almeida G., de Souza V., Sano S., Saccol M., Cohen M. (2012). Comparison of hip rotation range of motion in judo athletes with and without history of low back pain. Man. Ther..

[44] Tak I., Weerink M., Barendrecht M. (2020). Judokas with low back pain have lower flexibility of the hip-spine complex: A case-control study. Phys. Ther. Sport.

[45] Okada T., Nakazato K., Iwai K., Tanabe M., Irie K., Nakajima H. (2007). Body mass, nonspecific low back pain, and anatomical changes in the lumbar spine in judo athletes. J. Orthop. Sport. Phys. Ther..

[46] Kujala U.M., Taimela S., Oksanen A., Salminen J.J. (1997). Lumbar mobility and low back pain during adolescence. A longitudinal three-year follow-up study in athletes and controls. Am. J. Sports Med..

[47] Murray E., Birley E., Twycross-Lewis R., Morrissey D. (2009). The relationship between hip rotation range of movement and low back pain prevalence in amateur golfers: An observational study. Phys. Ther. Sport.

[48] Kujala U.M., Taimela S., Salminen J.J., Oksanen A. (1994). Baseline anthropometry, flexibility and strength characteristics and future low-back pain in adolescent athletes and nonathletes: A prospective one-year follow-up study. Scand. J. Med. Sci. Sports.

[49] Burdorf A., van der Steenhoven G.A., Tromp-Klaren E.G.M. (1996). A one-year prospective study on back pain among novice golfers. Am. J. Sports Med..

[50] Evans K., Refshauge K., Adam R., Aliprandi L. (2005). Predictors of low back pain in young elite golfers: A preliminary study. Phys. Ther. Sport.

[51] Terada K., Mullineaux D., Lanovaz J., Kato K., Clayton H. (2004). Electromyographic Activity of the Rider’s Muscles at Trot. Equine Comp. Exerc. Physiol..

[52] Terada K. (2000). Comparison of Head Movement and EMG Activity of muscles between advanced and novice horseback riders at different gaits. J. Equine Sci..

[53] McGill S. (2015). Low Back Disorders: Evidence-Based Prevention and Rehabilitation.

[54] Bussey M.D. (2010). Does the demand for asymmetric functional lower body postures in lateral sports relate to structural asymmetry of the pelvis?. J. Sci. Med. Sport.

[55] Gnat R., Saulicz E. (2008). Induced static asymmetry of the pelvis is associated with functional asymmetry of the lumbo-pelvo-hip complex. J. Manipulative Physiol. Ther..

[56] Symes D., Ellis R. (2009). A preliminary study into rider asymmetry within equitation. Vet. J..

[57] Gurney B. (2002). Leg length discrepancy. Gait Posture.

[58] Beattie P., Isaacson K., Riddle D.L., Rothstein J.M. (1990). Validity of derived measurements of leg-length differences obtained by use of a tape measure. Phys. Ther..

[59] McGuigan M.R., Al Dayel A., Tod D., Foster C., Newton R.U., Pettigrew S. (2008). Use of session rating of perceived exertion for monitoring resistance exercise in children who are overweight or obese. Pediatr. Exerc. Sci..

[60] Aguilar Cordero M.J., Ortegón Piñero A., Mur Villar N., Sánchez García J.C., García Verazaluce J.J., García I.G., Sánchez López A.M. (2014). Programas de actividad física para reducir sobrepeso y obesidad en niños y adolescentes: Revisión sistemática. Nutr. Hosp..

[61] Durall C., Udermann B., Johansen D., Gibson B., Reineke D., Reuteman P. (2009). The effects of preseason trunk muscle training on low-back pain occurrence in women collegiate gymnasts. J. Strength Cond. Res..

